# Stomatin-like protein 2 deficiency exacerbates adverse cardiac remodeling

**DOI:** 10.1038/s41420-023-01350-z

**Published:** 2023-02-14

**Authors:** Yuntao Hu, Hongwei Jiang, Yueyue Xu, Ganyi Chen, Rui Fan, Yifei Zhou, Yafeng Liu, Yiwei Yao, Renjie Liu, Wen Chen, Ke Zhang, Xin Chen, Rui Wang, Zhibing Qiu

**Affiliations:** 1 Department of Thoracic and Cardiovascular Surgery, Nanjing First Hospital, Nanjing Medical University, Jiangsu, China; 2 School of Medicine, Southeast University, Jiangsu, China; 3 Department of Thoracic and Cardiovascular Surgery, Changzhou Second People’s Hospital, Nanjing Medical University, Jiangsu, China

**Keywords:** Molecular biology, Cardiomyopathies, Heart failure

## Abstract

Myocardial fibrosis, oxidative stress, and autophagy both play key roles in the progression of adverse cardiac remodeling. Stomatin-like protein 2 (SLP-2) is closely related to mitochondrial function, but little is known about its role and mechanism in cardiac remodeling. We developed doxorubicin (Dox), angiotensin (Ang) II, and myocardial ischemia-reperfusion (I/R) injury induced cardiac remodeling model and Dox treated H9C2 cell injury model using SLP-2 knockout (SLP-2^-/-^) mice and H9C2 cells with low SLP-2 expression. We first examined cardiac functional and structural changes as well as levels of oxidative stress, apoptosis and autophagy. We found that SLP-2 deficiency leads to decreased cardiac function and promotes myocardial fibrosis. After Dox and Ang II treatment, SLP-2 deficiency further aggravated myocardial fibrosis, increased myocardial oxidative stress and apoptosis, and activated autophagy by inhibiting PI3K-Akt-mTOR signaling pathway, ultimately exacerbating adverse cardiac remodeling. Similarly, SLP-2 deficiency further exacerbates adverse cardiac remodeling after myocardial I/R injury. Moreover, we extracted cardiomyocyte mitochondria for proteomic analysis, suggesting that SLP-2 deficiency may be involved in myocardial I/R injury induced adverse cardiac remodeling by influencing ubiquitination of intramitochondrial proteins. In addition, the oxidative stress, apoptosis and autophagy levels of H9C2 cells with low SLP-2 expression were further enhanced, and the PI3K-Akt-mTOR signaling pathway was further inhibited under Dox stimulation. Our results suggest that SLP-2 deficiency promotes myocardial fibrosis, disrupts normal mitochondrial function, overactivates autophagy via PI3K-Akt-mTOR signaling pathway, affects the level of ubiquitination, leads to irreversible myocardial damage, and ultimately exacerbates adverse cardiac remodeling.

## Introduction

Cardiovascular disease is the greatest threat to human health around the world and is the leading cause of death in China [1, 2]. Cardiac remodeling is closely associated with a variety of cardiac diseases, characterized by progressive ventricular dilatation, myocardial hypertrophy, myocardial fibrosis, and deterioration of cardiac function [3]. The mechanisms of cardiac remodeling include changes in cardiomyocyte, such as redox injury, endoplasmic reticulum stress, and cardiomyocyte autophagy, as well as phenotypic changes in other non-cardiomyocytes such as fibroblasts, endothelial cells, and inflammatory cells [4–6]. Myocardial fibrosis, as one of the key factors in cardiac remodeling, has received extensive attention in recent years. Myocardial fibrosis is characterized by activation of cardiac fibroblasts [7], which is mainly manifested by disordered arrangement and excessive deposition of myocardial interstitial and perivascular collagen fibers, resulting in cardiac systolic and diastolic dysfunction, arrhythmia, and heart failure [8–10]. Cardiac fibroblasts are highly plastic and can be converted into cardiac myofibroblasts under various injury or stress conditions [11]. Cardiac myofibroblasts can cause myocardial fibrosis by synthesizing excessive extracellular matrix, such as collagen I and III, resulting in cardiomyocyte injury and death, and eventually develop into impaired cardiac function [10].

Autophagy is a classical form of cell death resulting in degradation of cytoplasmic contents after stimulation and injury, responsible for the removal of potentially toxic cytoplasmic protein aggregates and damaged organelles [12–14]. Autophagy is a strictly regulated mechanism of lysosome degradation, which is crucial for cell survival, homeostasis, and function [15]. Autophagy is activated excessively under some conditions, causing uncontrolled cell degradation and death [16]. Mitochondria, as the center of energy metabolism, play an important role in autophagy [17]. Abnormal activation of mitophagy will lead to mitochondrial depletion, resulting in energy metabolism disorder and reactive oxygen species (ROS) accumulation, and eventually lead to cell senescence and death [18]. Overactivation of autophagy in cardiomyocyte leads to excessive digestion and degradation of proteins and organelles [16]. Previous studies have shown that ROS can aggravates myocardial I/R injury by activating autophagy [19]. Inhibition of autophagic initiation or flux prevents cell death and alleviates cardiac injury or dysfunction in some conditions [20, 21]. A study shows that autophagy can convert cardiac fibroblasts into cardiac myofibroblasts, suggesting that autophagy may be involved in the occurrence and development of myocardial fibrosis [22]. Therefore, autophagy may be an important target for improving myocardial fibrosis, alleviating myocardial oxidative stress injury and reversing adverse cardiac remodeling.

SLP-2 is a mitochondrial inner membrane protein, which is abundant in skeletal muscle and heart [23], and up-regulated in most tumor cells [24, 25]. Previous studies have shown that SLP-2 plays a critical role in protecting mitochondrial function. Up-regulation of SLP-2 expression increases mitochondrial oxygen consumption and ATP production, as well as mitochondrial cardiolipin synthesis, which increases mitochondrial membrane formation and biogenesis, and promotes the assembly of respiratory supercomplexes [26]. Our previous research suggests that overexpression of SLP-2 reduces cardiomyocyte apoptosis, mitochondrial injury, and myocardial I/R injury [27]. A study on gastric cancer shows that silencing SLP-2 expression can induce apoptosis and autophagy in cancer cells [28]. At present, the mechanism of SLP-2 in myocardial fibrosis and cardiomyocyte autophagy remains unclear.

Therefore, we explored for the first time the mechanism that SLP-2 deficiency can mediate myocardial fibrosis and adverse cardiac remodeling by regulating the PI3K-Akt-mTOR signaling pathway to enhance autophagy, which will play a vital role in alleviating myocardial fibrosis, cardiac remodeling and improving the prognosis of patients with end-stage heart disease.

## Results

### SLP-2 deficiency induces cardiac function decline and promotes myocardial fibrosis

**Fig. 1:**
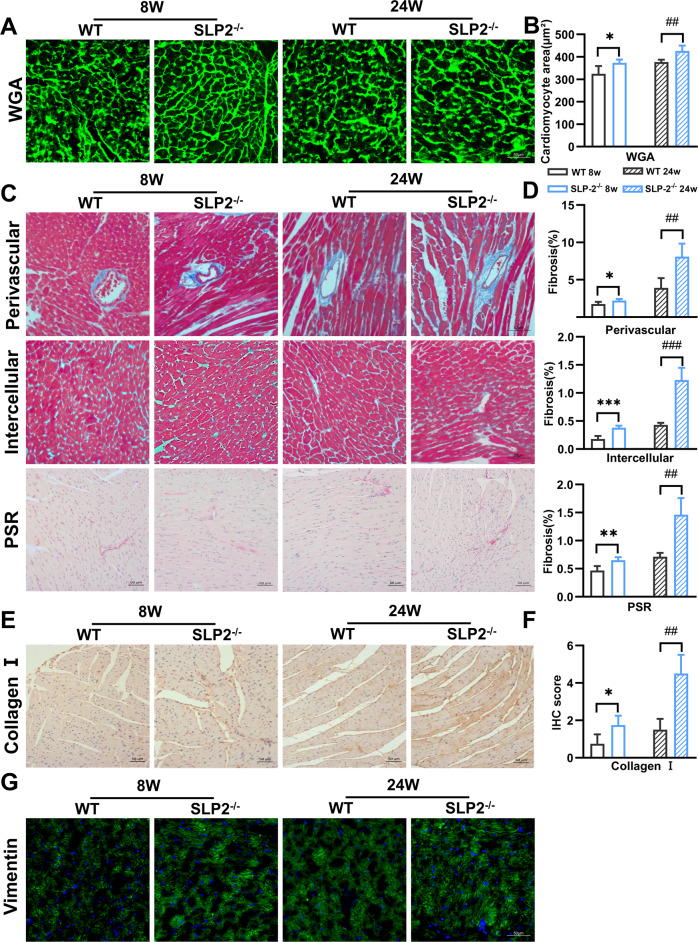
SLP-2 deficiency leads to cardiac hypertrophy and promotes myocardial fibrosis. **A**–**G** Heart representative images of WGA staining, mean cardiomyocyte area, Masson and PSR staining, percentage of fibrosis, IHC staining of collagen I, and IF staining of Vimentin in WT and SLP-2^–/–^ mice at 8w and 24w. (*n* = 4 per group, *P < 0.05, **P < 0.01, ***P < 0.001 vs. WT mice 8w, #*P* < 0.05, ##*P* < 0.01, ###*P* < 0.001 vs. WT mice 24w, student’s *t* test).

To comprehend the function of SLP-2 in the heart, we constructed SLP-2 whole-body knockout mice. We utilized male mice aged 6–24 weeks to examine the role of SLP-2 in cardiac remodeling. The expression of SLP-2 in the heart of SLP-2^–/–^ mice decreased by nearly 90% compared with WT mice. We found that when SLP-2^–/–^ mice aged, heart gradually expanded larger and heavier, and abnormal cardiac remodeling eventually developed into heart failure (Fig. S1). There was no significant difference in heart weight/tibia length (HW/TL) between SLP-2^–/–^ mice and WT mice at 6 weeks old, but increased by 18.4%, 18.3%, 21.1% and 19.94% compared with WT mice at 8 weeks, 10 weeks, 16 weeks and 24 weeks, respectively (Fig. S1B). Similarly, echocardiography showed that there was no difference in EF and FS at 6 weeks, but EF and FS in SLP-2^–/–^ mice began to decrease at 8 weeks, and at 24 weeks, EF decreased by 25%, FS decreased by 23% (Fig. S1C, D). The LV mass, IVS thickness, and LVPW thickness of SLP-2^–/–^ mice were also significantly increased compared with WT mice (Fig. S1D), which was further demonstrated by HE staining (Fig. S1E). By WGA staining, we found that the cardiomyocyte area in SLP-2^–/–^ group was significantly larger than WT group at 8w or 24w (Fig. 1A, B). Masson and PSR staining showed that the degree of fibrosis in SLP-2^–/–^ mice increased at 8 weeks and significantly aggravated at 24 weeks (Fig. 1C, D). The main feature of myocardial fibrosis is the deposition of extracellular matrix proteins, such as collagen I and III. The results of IHC staining showed that the expression of collagen I in SLP-2^–/–^ group was significantly higher than WT group (Fig. 1E, F). Cardiac fibroblasts play a key role in the occurrence and development of cardiac fibrosis. We found that the number of cardiac fibroblasts in SLP-2^–/–^ mice increased significantly by IF staining (Fig. 1G). These results suggest that SLP-2 deficiency leads to cardiomyocyte hypertrophy and myocardial fibrosis.

### SLP-2 deficiency exacerbates Dox-induced DCM phenotype

**Fig. 2:**
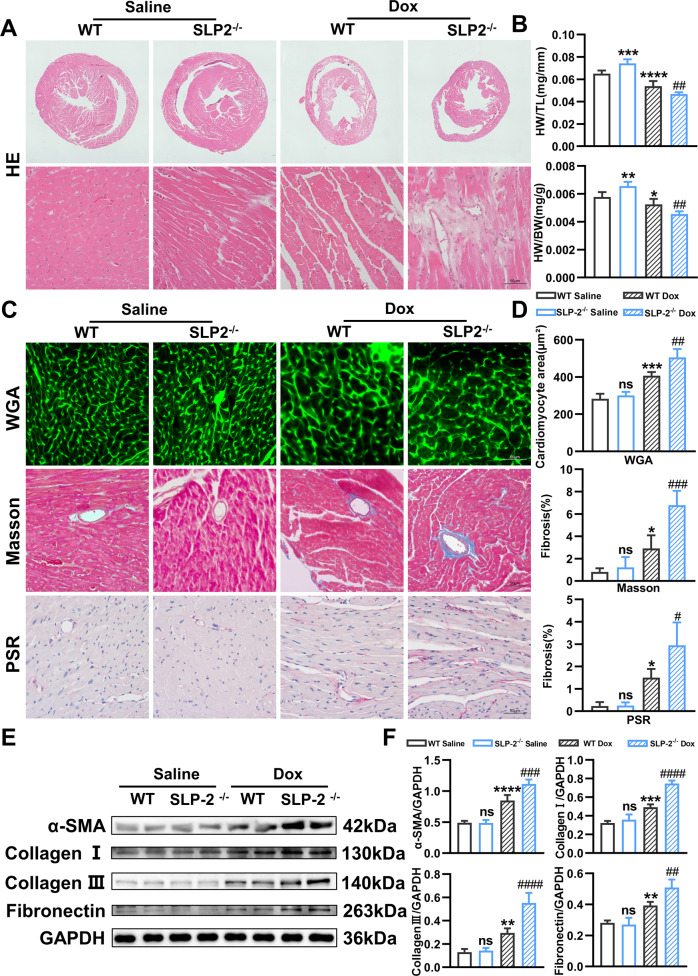
SLP-2 deficiency exacerbates Dox-induced DCM phenotype. **A** Representative images of HE staining in WT and SLP-2^–/–^ mice hearts after saline or dox treatment. **B** HW/TL and heart weight/body weight in WT and SLP-2^–/–^ mice after saline or Dox treatment. **C**–**F** Representative images of WGA, Masson and PSR staining, cardiomyocyte area and percentage of fibrosis, and expression of α-SMA, collagen I, collagen III and Fibronectin, and quantitative analysis in WT and SLP-2^–/–^ mice hearts after saline or Dox treatment. (*n* = 4 per group, *P < 0.05, **P < 0.01, ***P < 0.001, ****P < 0.0001 vs. WT Saline group, #*P* < 0.05, ##*P* < 0.01, ###*P* < 0.001, ####*P* < 0.0001 vs. WT Dox group, 1-way ANOVA, Tukey test).

We further investigated the role of SLP-2 in the progression of Dox-induced dilated cardiomyopathy (DCM). We first tested the serum biochemical indexes of hepatic and renal function in mice, and the results indicated that SLP-2 deficiency did not exacerbate the hepatic and renal function injury in mice under saline or Dox treatment (Fig. S2A, B). We could rule out the adverse cardiac remodeling secondary to damaged hepatic and renal function caused by SLP-2 deficiency. HE staining and echocardiography showed that compared with the saline group, the heart volume was significantly reduced, the ventricular wall and septum were significantly thinner, the inner diameter of the ventricle was significantly increased, the ventricular cavity was significantly expanded, and the cardiac function was significantly decreased in the Dox group. The DCM phenotype of SLP-2^-/-^ Dox group was more obvious than WT Dox group (Fig. 2A, B, Fig. S3A, B). WGA staining showed no significant difference in cardiomyocyte area between the two saline groups, while SLP-2 deficiency exacerbated cardiomyocyte hypertrophy under Dox treatment (Fig. 2C, D). Masson and PSR staining indicated that the degree of myocardial perivascular and intercellular fibrosis was significantly aggravated in Dox group (Fig. 2C, D), and IHC staining indicated that the expression of myocardial fibrosis markers (a-SMA and collagen III) was significantly increased in Dox group (Fig. S3C). Similarly, Western blot analysis showed that the expression of a-SMA, collagen I, collagen III and Fibronectin in Dox group were significantly higher than saline group (Fig. 2E, F). More importantly, SLP-2 deficiency significantly aggravated the above-mentioned levels of myocardial fibrosis under Dox treatment (Fig. 2C–F and S3C). These results indicate that SLP-2 deficiency exacerbates Dox-induced DCM phenotype.

### SLP-2 deficiency increases the levels of oxidative stress and apoptosis in Dox-induced DCM

**Fig. 3:**
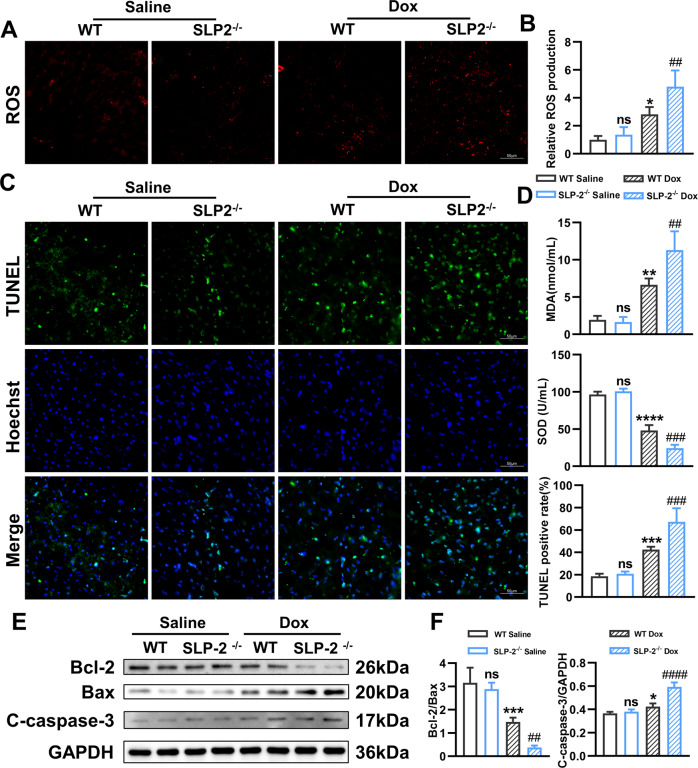
SLP-2 deficiency increases the levels of oxidative stress and apoptosis in Dox-induced DCM. **A**–**C** Heart representative images of ROS staining and relative ROS production, and TUNEL staining in WT and SLP-2^-/-^ mice after saline or Dox treatment. **D** Concentration of MDA and SOD in serum, and TUNEL positive rate in WT and SLP-2^–/–^ mice after saline or Dox treatment. **E**, **F** Representative images of expression of Bcl-2, Bax and C-caspase-3, and quantitative analysis in WT and SLP-2^–/–^ mice hearts after saline or Dox treatment. (*n* = 4 per group, *P < 0.05, **P < 0.01, ***P < 0.001, ****P < 0.0001 vs. WT Saline group, #*P* < 0.05, ##*P* < 0.01, ###*P* < 0.001, ####*P* < 0.0001 vs. WT Dox group, 1-way ANOVA, Tukey test).

SLP-2 is located in mitochondrial inner membrane. To further explore the effect of SLP-2 deficiency on mitochondrial damage and oxidative stress levels in DCM, we measured ROS production in cardiomyocytes and MDA and SOD levels in serum of mice. The results showed that compared with saline group, ROS, MDA and apoptosis levels (TUNEL, Bax, Cleaved-caspase-3) were significantly increased, SOD level and the expression of anti-apoptotic protein (Bcl-2) were significantly decreased in Dox group. Moreover, SLP-2 deficiency further increases the level of oxidative stress and apoptosis in Dox group (Fig. 3A–F). These results indicate that SLP-2 deficiency aggravates Dox-induced DCM by causing mitochondrial function impairment, myocardial oxidative stress injury and apoptosis.

### SLP-2 deficiency exacerbates DCM progression by regulating PI3K-Akt-mTOR signaling pathway

**Fig. 4:**
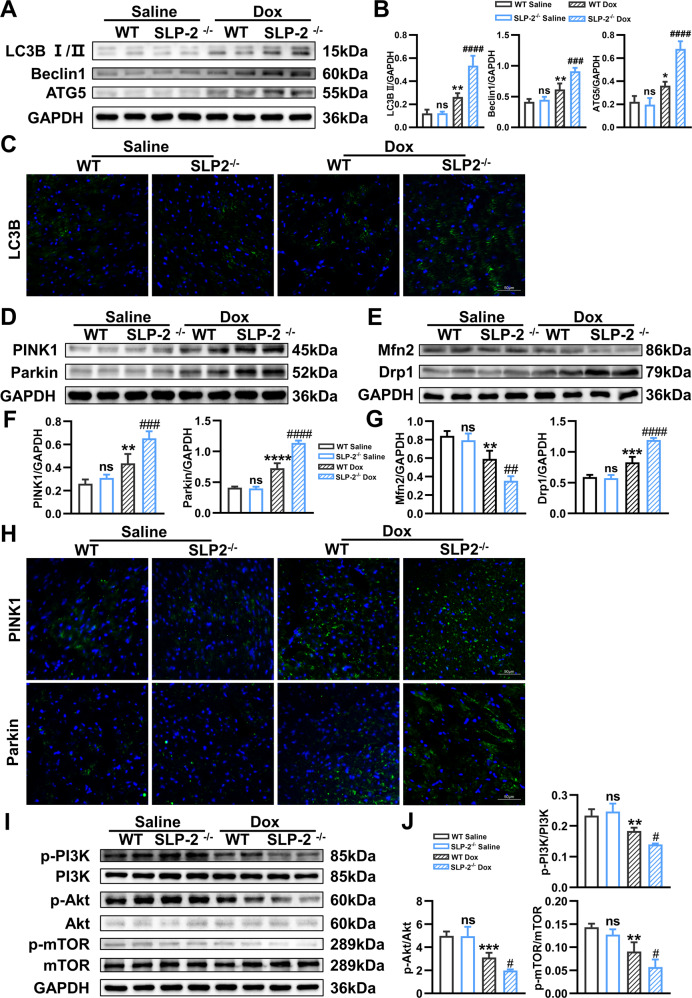
SLP-2 deficiency exacerbates DCM progression by regulating PI3K-Akt-mTOR signaling pathway. **A**–**C** Representative images of expression of LC3B I/II, Beclin1 and ATG5, and quantitative analysis, and IF staining of LC3B in WT and SLP-2^–/–^ mice hearts after saline or Dox treatment. **D**, **F** and **H**, Representative images of expression of PINK1 and Parkin, and quantitative analysis, and IF staining of PINK1 and Parkin in WT and SLP-2^–/–^ mice hearts after saline or Dox treatment. **E**, **G** Representative images of expression of Mfn2 and Drp1, and quantitative analysis in WT and SLP-2^–/–^ mice hearts after saline or Dox treatment. **I**, **J** Representative images of expression of p-PI3K, PI3K, p-Akt, Akt, p-mTOR and mTOR, and quantitative analysis in WT and SLP-2^–/–^ mice hearts after saline or Dox treatment. (*n* = 4 per group, *P < 0.05, **P < 0.01, ***P < 0.001, ****P < 0.0001 vs. WT Saline group, #*P* < 0.05, ##*P* < 0.01, ###*P* < 0.001, ####*P* < 0.0001 vs. WT Dox group, 1-way ANOVA, Tukey test).

We further explored the effect of SLP-2 deficiency on autophagy in DCM. Western blot analysis showed that the expression of autophagy-associated proteins (LC3B II, Beclin1 and ATG5) in SLP-2^–/–^ Dox group was significantly higher than WT Dox (Fig. 4A, B). The area of LC3B positive region in IF staining verified the above result (Fig. 4C). The expressions of mitophagy-associated proteins (PINK1 and Parkin) were increased after Dox treatment, and even more significantly after SLP-2 deficiency (Fig. 4D, F). The IF staining results verified the above result (Fig. 4H). Mitochondrial fusion and fission are closely related to mitophagy. Our results showed that the expression of mitochondrial fusion protein Mfn2 decreased, fission protein Drp1 increased after Dox treatment, and SLP-2 deficiency exacerbated the change trend (Fig. 4E, G). In addition, there was no significant difference in all the above indexes between the two saline groups. We then detected the expression of PI3K-Akt-mTOR signaling pathway, western blot analysis showed that the protein ratios of p-PI3K/PI3K, p-Akt/Akt, p-mTOR/mTOR in Dox group were significantly lower than saline group, and SLP-2 deficiency further reduced all ratios, suggesting that SLP-2 deficiency inhibited PI3K-Akt-mTOR signaling pathway, leading to excessive activation of autophagy (Fig. 4I, J). These results indicate that SLP-2 deficiency exacerbates the DCM progression by regulating PI3K-Akt-mTOR signaling pathway to promote autophagy.

### SLP-2 deficiency exacerbates Ang II-induced cardiac remodeling

**Fig. 5:**
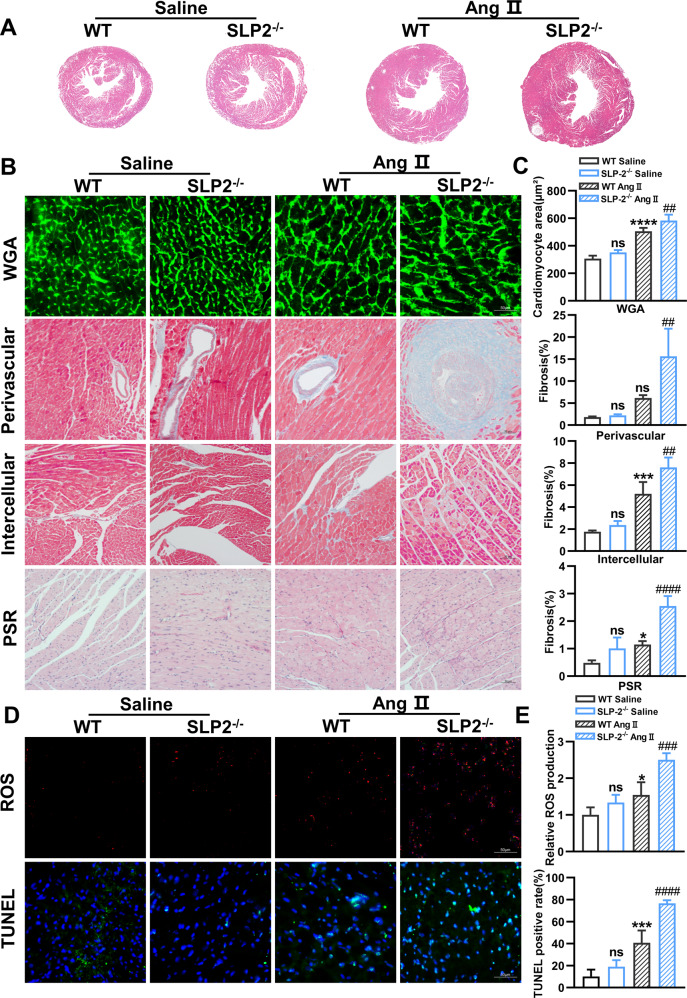
SLP-2 deficiency exacerbates Ang II-induced cardiac remodeling. **A**–**E** Representative images of HE, WGA, Masson and PSR staining, and cardiomyocyte area and percentage of fibrosis, and ROS and TUNEL staining, and relative ROS production and TUNEL positive rate in WT and SLP-2^–/–^ mice hearts after saline or Ang II treatment. (*n* = 4 per group, *P < 0.05, **P < 0.01, ***P < 0.001, ****P < 0.0001 vs. WT Saline group, #*P* < 0.05, ##*P* < 0.01, ###*P* < 0.001, ####*P* < 0.0001 vs. WT Ang II group, 1-way ANOVA, Tukey test).

We further investigated whether SLP-2 affects Ang II-induced cardiac remodeling. HE and WGA staining showed that SLP-2 deficiency significantly aggravated Ang II-induced cardiomyocyte hypertrophy (Fig. 5A–C). Masson and PSR staining showed that the degree of myocardial fibrosis in SLP-2^–/–^ Ang II group was significantly higher than WT Ang II group (Fig. 5B, C). Consistent with the results after Dox stimulation, SLP-2 deficiency further aggravated the increase of ROS production and TUNEL positive rate in cardiomyocytes under Ang II treatment (Fig. 5D, E). Then we detected the expression of autophagy-associated proteins and PI3K-Akt-mTOR signaling pathway. The results showed that the expression of LC3B further increased in SLP-2 deficiency, while the ratios of p-PI3K/PI3K, p-Akt/Akt and p-mTOR/mTOR decreased significantly under Ang II treatment (Fig. S4A, B). These results indicate that SLP-2 deficiency promotes autophagy by regulating PI3K-Akt-mTOR signaling pathway and plays a vital role in Ang II-induced cardiac remodeling.

### SLP-2 deficiency exacerbates myocardial I/R injury by affecting the level of ubiquitination of intramitochondrial proteins

**Fig. 6:**
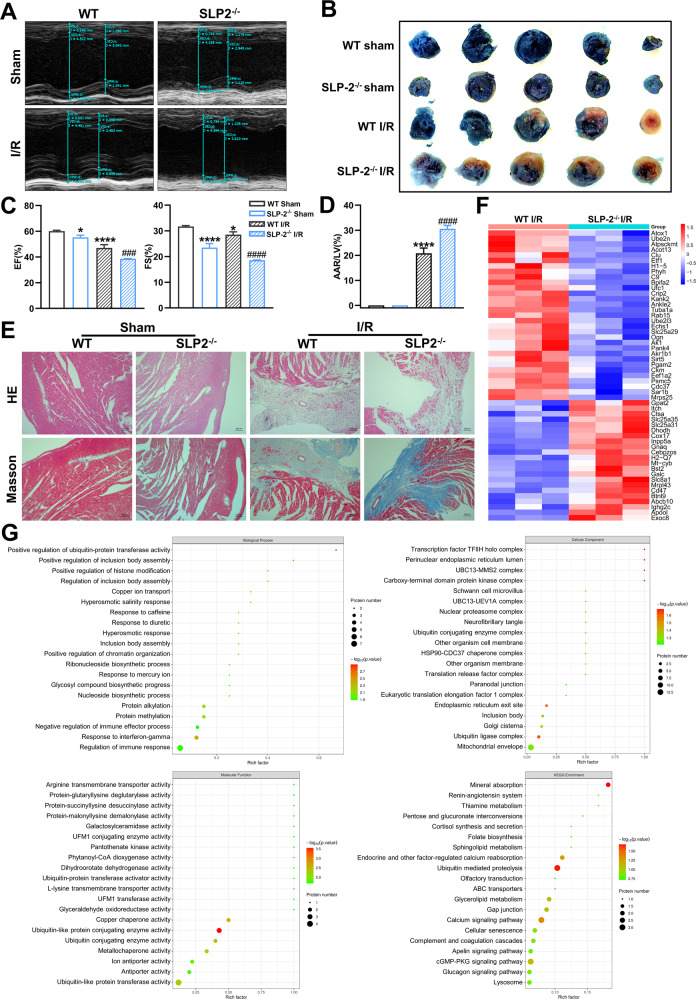
SLP-2 deficiency exacerbates myocardial I/R injury by affecting the level of ubiquitination of intramitochondrial proteins. **A**, **C** Representative echocardiographs and echocardiographic parameters in WT and SLP-2^–/–^ mice after sham or myocardial I/R injury. **B**, **D** Cross-sections of left ventricles (LV) stained with Evans Blue/TTC and statistical graph of AAR/LV. **E** Heart representative images of HE and Masson staining in WT and SLP-2^–/–^ mice after sham or myocardial I/R injury. **F**, **G** Heat map, gene ontology analysis and KEGG analysis of differentially expressed proteins in WT and SLP-2^–/–^ mice were obtained by mitochondrial proteomics after myocardial I/R injury. (*n* = 3 per group, *P < 0.05, **P < 0.01, ***P < 0.001, ****P < 0.0001 vs. WT Saline group, #*P* < 0.05, ##*P* < 0.01, ###*P* < 0.001, ####*P* < 0.0001 vs. WT Ang II group, 1-way ANOVA, Tukey test).

Previously, we confirmed that SLP-2 deficiency exacerbates adverse cardiac remodeling, but both Dox and Ang II stimulation are the procession of long-term chronic injury. Then we construct a model of acute myocardial I/R injury to verify whether SLP-2 is involved in cardiac remodeling caused by acute injury. By echocardiography, we found that the cardiac function of SLP-2^–/–^ mice decreased significantly (Fig. 6A, C). We measured the area at risk (AAR) and LV areas in mice by Evans blue/TTC staining. The result showed that SLP-2 deficiency further increased the AAR/LV ratio and aggravated the myocardial I/R injury (Fig. 6B, D). HE staining showed that the accumulation of inflammatory cells was more obvious, the infiltration depth was deeper, and the myocardial infarction area was larger in SLP-2^–/–^ mice after myocardial I/R injury (Fig. 6E). Masson staining showed that myocardial fibrosis was significantly aggravated after myocardial I/R injury in SLP-2^–/–^ mice (Fig. 6E). These results suggest that SLP-2 deficiency exacerbates the adverse cardiac remodeling caused by myocardial I/R injury. To explore how SLP-2 is involved in myocardial I/R injury, we extracted cardiomyocyte mitochondria for proteomics from WT and SLP-2^–/–^ mice with ischemia for 45 min followed by reperfusion for 6 h. Through the functional analysis of 53 differentially expressed proteins obtained from proteomics (Fig. 6F), we found that ubiquitination was involved in biological process, cellular component and molecular function of gene ontology analysis and KEGG enrichment. ubiquitination is closely related to apoptosis, autophagy and mitochondrial quality control system [29–31]. We speculate that SLP-2 deficiency may affect the level of ubiquitination of intramitochondrial proteins, then promote apoptosis and autophagy, damage the mitochondrial quality control system, and finally aggravate myocardial I/R injury (Fig. 6G).

### Low SLP-2 expression leads to increased apoptosis and mitochondrial dysfunction in H9C2 cells under Dox stimulation

Western blot analysis showed that the expression of SLP-2 was significantly decreased in H9C2 cells transfected with siRNA1, siRNA2 and siRNA3, and siRNA2 SLP-2 most effectively reduced SLP-2 expression (Fig. S5A, B). We selected siRNA2 SLP-2 for cell transfection in the follow-up experiment.

**Fig. 7:**
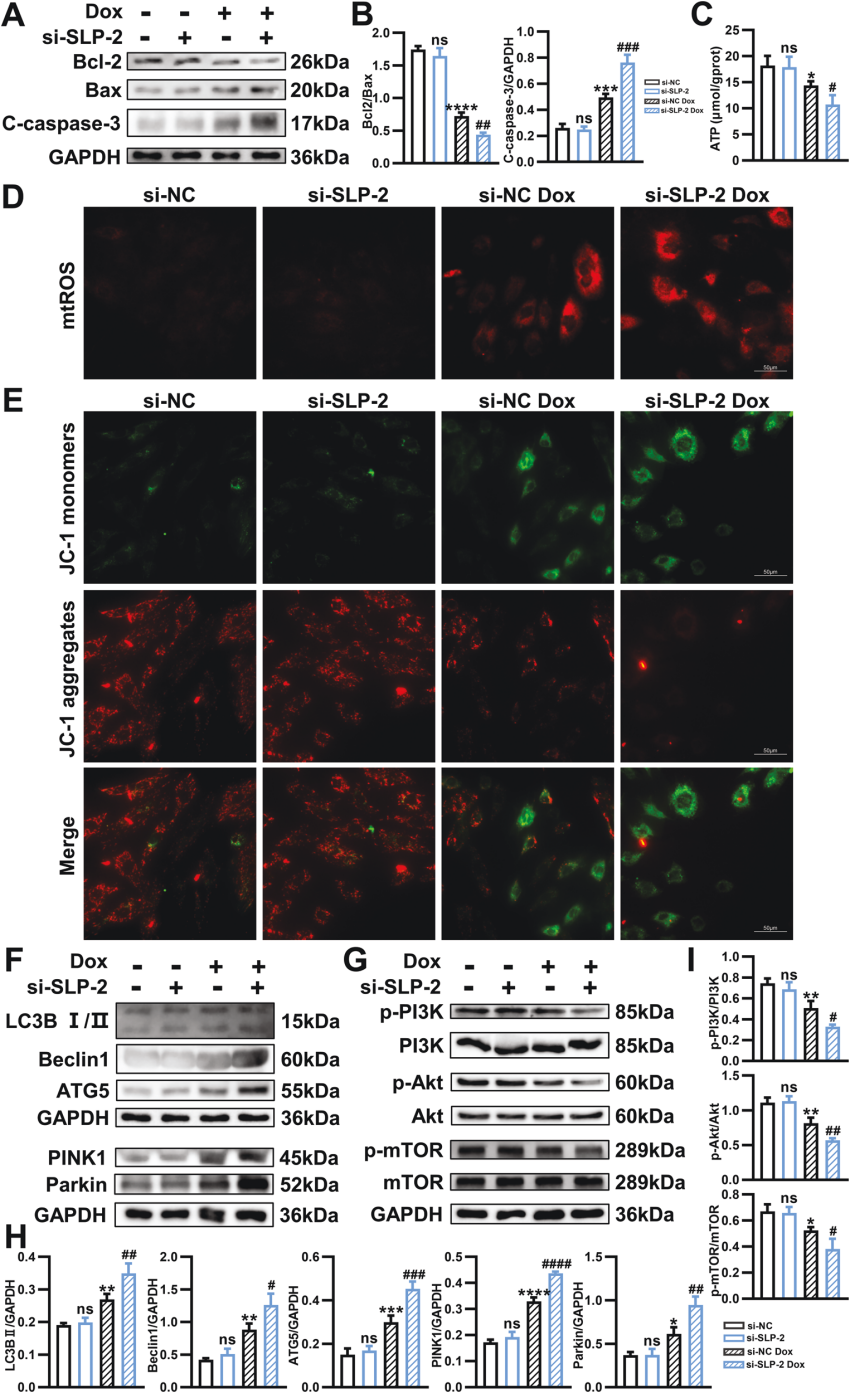
Low SLP-2 expression aggravates H9C2 cells injury under Dox stimulation by promoting mitochondrial dysfunction and autophagy. **A**, **B** Representative images of expression of Bcl-2, Bax and C-caspase-3, and quantitative analysis in different groups of H9C2 cells. **C** ATP levels in different groups of H9C2 cells. **D** Representative images of mitochondrial peroxide staining in different groups of H9C2 cells. **E** Representative fluorescence images of JC-1 staining in different groups of H9C2 cells. **F**, **H** Representative images of expression of LC3B, Beclin1, ATG5, PINK1 and Parkin, and quantitative analysis in different groups of H9C2 cells. **G**, **I** Representative images of expression of p-PI3K, PI3K, p-Akt, Akt, p-mTOR and mTOR, and quantitative analysis in different groups of H9C2 cells. (*n* = 3–4 per group, *P < 0.05, **P < 0.01, ***P < 0.001, ****P < 0.0001 vs. si-NC group, #*P* < 0.05, ##*P* < 0.01, ###*P* < 0.001, ####*P* < 0.0001 vs. si-NC Dox group, 1-way ANOVA, Tukey test).

We then evaluated the effects of low SLP-2 expression on apoptosis and mitochondrial function of H9C2 cells. The results showed that low SLP-2 expression significantly increased the level of apoptosis (TUNEL, Bax/Bcl-2, C-caspase-3), decreased ATP content and mitochondrial membrane potential, increased mtROS production under Dox stimulation (Fig. 7A–E, S5C, D). These results indicate that low SLP-2 expression leads to increased apoptosis and mitochondrial dysfunction in H9C2 cells under Dox stimulation.

### Low SLP-2 expression aggravates H9C2 cells injury under Dox stimulation by regulating PI3K-Akt-mTOR signaling pathway to promote autophagy

We further examined the effect of low SLP-2 expression on autophagy of H9C2 cells. The results showed that the expression of autophagy-associated proteins (LC3B II, Beclin1 and ATG5) and mitophagy-associated proteins (PINK1 and Parkin) in si-SLP-2 Dox group was significantly higher than si-NC Dox group, and there was no significant difference between si-NC group and si-SLP-2 group (Fig. 7F, H), which was consistent with previous in vivo results.

3-MA is a commonly used autophagy inhibitor. After pretreatment of H9C2 cells with 3-MA, the expression of autophagy-associated proteins (LC3B II, Beclin1 and ATG5) decreased significantly in si-SLP-2 Dox group (Fig. S6A, B), while the mitochondrial membrane potential increased and mtROS production decreased (Fig. S6C, D), suggesting that H9C2 cells injury caused by low SLP-2 expression under Dox stimulation was alleviated after inhibition of autophagy.

We further explored the expression of PI3K-Akt-mTOR signaling pathway. Western blot analysis showed that the ratios of p-PI3K/PI3K, p-Akt/Akt and p-mTOR/mTOR in H9C2 cells decreased significantly after Dox stimulation, and low SLP-2 expression further reduced the above ratios. These results suggest that low SLP-2 expression suppresses the PI3K-Akt-mTOR signaling pathway of H9C2 cells under Dox stimulation, activates autophagy and leads to H9C2 cells injury.

## Discussion

Cardiovascular disease has become the primary disease that threatens human life [1, 2]. Most cardiovascular diseases develop into irreversible cardiac remodeling and heart failure at the end stage. The mechanism of cardiac remodeling is very complex, which is the result of the comprehensive action of a variety of pathological factors [32, 33]. The main factors that promote the development of cardiac remodeling include myocardial fibrosis, excessive deposition of extracellular matrix, oxidative stress and apoptosis [34, 35]. Up to now, the progress of improving and alleviating cardiac remodeling is still the focus and difficulty in the treatment of cardiovascular disease [32]. Our study focused on the role of SLP-2 in different stimulus-induced cardiac remodeling or H9C2 cell injury under Dox stimulation, aiming to explore the mechanism of the development of cardiac remodeling in order to seek to improve and alleviate the progression of cardiac remodeling.

Excessive deposition of extracellular matrix and myocardial fibrosis are involved in cardiac remodeling [32, 35, 36]. Under the stimulation of pathological stress, cardiac fibroblasts are transformed into myofibroblasts, which can secrete extracellular matrix components such as collagen I, collagen III and fibronectin. Excessive deposition of extracellular matrix and fibrosis lead to myocardial tissue disorder, cardiac systolic and diastolic dysfunction, and eventually develop into cardiac remodeling [32]. In our study, the degree of myocardial fibrosis and the expression of α-SMA in cardiac fibroblasts in SLP-2^–/–^ group were higher than WT group after Dox treatment, which indicated that SLP-2 deficiency promoted the transformation of cardiac fibroblasts into myofibroblasts. At the same time, the expression of collagen I, collagen III and fibronectin in SLP-2^–/–^ Dox group was significantly higher than WT Dox group. HE staining and echocardiography showed that compared with WT Dox group, the ventricular cavity of SLP-2^–/–^ Dox group was further dilated, the ventricular wall and IVS were thinner, and the cardiac function was significantly decreased. These results indicate that SLP-2 deficiency aggravates extracellular matrix deposition and myocardial fibrosis, and promotes the progression of adverse cardiac remodeling.

Excessive accumulation of ROS can lead to oxidative stress damage and damage to lipids, proteins and DNA, resulting in cell death [37]. Mammalian cardiomyocytes almost completely lose their ability to proliferate in adulthood, and will be replaced by scar tissue after cardiomyocyte death, resulting in myocardial structural and electrophysiological dysfunction, eventually develop into cardiac remodeling [38, 39]. Previous studies have shown that Dox can mediate mitochondrial dysfunction and aggravate oxidative stress injury and cardiomyocyte apoptosis [40]. SLP-2, as a mitochondrial inner membrane protein, is closely related to oxidative stress [26]. A study showed that overexpression of SLP-2 significantly increased the cell viability and decreased apoptosis and ROS production in neurons injured by oxygen-glucose deprivation /reoxygenation, while inhibition of SLP-2 showed the opposite effect [41]. Consistent with this, our study shows that SLP-2 deficiency further aggravates the damage effect of Dox, the levels of ROS, MDA and SOD in myocardium are significantly increased, and the decrease of Bcl-2/Bax ratio and the increase of Cleaved-caspase-3 level indicate that myocardial apoptosis is further increased. Therefore, SLP-2 deficiency may aggravate the progression of cardiac remodeling by increasing myocardial oxidative stress injury and apoptosis.

A great deal of evidence shows that autophagy is closely related to the progression of a variety of heart diseases, such as coronary artery disease, DCM, hypertrophic cardiomyopathy and myocardial I/R injury [42–45]. Studies suggest that excessive autophagy leads to DCM, while inhibition of autophagy can reduce myocardial infarction area, inhibit cardiac remodeling and improve cardiac function [43, 46, 47]. Studies have shown that excessive mitochondrial fission can lead to excessive mitophagy, which exacerbates cardiac remodeling, and overexpression of SLP-2 can significantly increase the content of mitochondrial fusion protein Mfn2, thereby reducing mitochondrial fission and oxidative stress injury [48–51]. Silencing SLP-2 can inhibit gastric cancer cell proliferation and induce apoptosis and autophagy via ANXA2/ β-catenin signal pathway [28]. In our study, the expression levels of LC3B II, Beclin1 and ATG5 in SLP-2^–/–^ Dox group were significantly higher than WT Dox group, suggesting that SLP-2 deficiency aggravated cardiomyocyte autophagy induced by Dox. Then we further explored the effect of SLP-2 deficiency on mitophagy in DCM. In SLP-2^–/–^ Dox group, the expression of mitophagy-related proteins PINK1 and Parkin and mitochondrial fission protein Drp1 were significantly increased, while the mitochondrial fusion protein Mfn2 was significantly decreased. These evidences suggest that SLP-2 deficiency promotes excessive autophagy of cardiomyocytes, causing abnormal mitochondrial fission and excessive mitophagy, and ultimately exacerbating the progression of Dox-induced adverse cardiac remodeling.

PI3K-Akt-mTOR signaling pathway is closely related to a variety of physiological activities and is a well-known pathway involved in autophagy regulation in mammals [52]. Phosphoinositide 3-kinase (PI3K) regulates cell signal transduction, energy metabolism and cell cycle [53, 54]. Akt known also as protein kinase B is regulated by PI3K and is mainly involved in apoptosis, autophagy and cell cycle regulation, while Akt can activate its downstream mammalian target of rapamycin (mTOR) [55]. mTOR is relatively conservative in evolution. It can integrate a variety of extracellular signals such as nutrition, energy and growth factors, participate in a variety of biological processes of transcription, translation and ribosome synthesis, and play an important role in cell growth and metabolism, apoptosis and autophagy [56–58]. One study showed that the activation of PI3K-Akt-mTOR signaling pathway can promote mitochondrial fusion and suppress mitochondrial fission, thus attenuating ischemia-reperfusion injury in diabetic cardiomyopathy [59]. As far as we know, the relationship between PI3K-Akt-mTOR signaling pathway, cardiomyocyte autophagy, and cardiac remodeling is unclear. In our study, we found that SLP-2 deficiency inhibits PI3K-Akt-mTOR signaling pathway in mouse heart under Dox treatment, promotes oxidative stress injury, apoptosis and mitochondrial dysfunction, leads to excessive activation of autophagy, and eventually develops into adverse cardiac remodeling.

Ang II can increase cardiac preload and postload, cause changes in myocardial morphology and function, increase extracellular matrix deposition and cardiac fibroblasts activation, and promote myocardial oxidative stress injury [60–62]. In our study, SLP-2 deficiency induced cardiomyocyte hypertrophy, promoted myocardial fibrosis, and significantly increased ROS production and apoptosis levels under Ang II stimulation. Similarly, SLP-2 deficiency further inhibits PI3K-Akt-mTOR signaling pathway and leads to increased autophagy of cardiomyocytes under Ang II stimulation, which is consistent with the results in Dox-induced cardiac remodeling, suggesting that SLP-2 deficiency leads to excessive activation of autophagy, which induces cardiomyocyte injury in mice and ultimately exacerbates adverse cardiac remodeling.

Myocardial I/R injury can cause acute cardiac injury in a short time. Complications of myocardial I/R injury can lead to adverse cardiac remodeling, including inflammation, activation of apoptosis, loss of cardiomyocytes and so on, and then develop into heart failure [63, 64]. Similarly, in our study, SLP-2 deficiency aggravated myocardial I/R injury and myocardial fibrosis, further decreased cardiac function, and eventually led to adverse cardiac remodeling. We further extracted the mitochondrial proteins of mouse cardiomyocytes for proteomics and analyzed the function of the differentially expressed proteins. The results showed that SLP-2 deficiency affected the ubiquitination of intramitochondrial proteins. ubiquitination is widely involved in the processes of apoptosis and autophagy. We speculate that SLP-2 deficiency regulates autophagy by affecting the level of ubiquitination, which aggravates cardiac remodeling after I/R injury. The specific mechanism remains to be verified by further research.

Finally, by silencing SLP-2 expression in H9C2 cells, we verified that low SLP-2 expression leads to increased apoptosis level and mitochondrial dysfunction of H9C2 cells, and promotes autophagy via PI3K-Akt-mTOR signaling pathway under Dox stimulation, which ultimately leads to H9C2 cells injury. After using autophagy inhibitor 3-MA to inhibit autophagy, the damaged mitochondrial membrane potential was restored and the production of mitochondrial ROS was decreased, which confirmed that excessive autophagy induced by low SLP-2 expression played an important role in the injury of H9C2 cells.

In summary, our research revealed for the first time that SLP-2 is closely related to the development of adverse cardiac remodeling. Pathological cardiac remodeling eventually develops into cardiac ejection dysfunction and heart failure, the latter is the main cause of death in the population. Therefore, it is necessary to explore how to improve and reverse adverse cardiac remodeling. Our research shows that SLP-2 deficiency exacerbates the progression of cardiac remodeling, so how to promote the expression of SLP-2 may be a vital direction of cardiac remodeling therapy in the future.

## Materials and methods

### Animals and experimental models

SLP-2^–/–^ and wild-type (C57BL/6, WT) male mice (6–24 weeks old) were purchased from GemPharmatech, Inc. (Nanjing, China) and underwent cultivation in the Model Animal Research Center of Nanjing University (Nanjing, China). All mice were housed in pathogen-free cages under a 12-hour light/12-hour dark cycle, controlled room temperature and given freely available diet and water. All animal experiments were approved by the Ethics Committee of Experimental Animal of Nanjing First Hospital, Nanjing Medical University.

All mice (22–25 g) were randomly divided into WT control, SLP-2^–/–^ control and corresponding experimental groups. We construct Dox-induced and Ang II-induced cardiac remodeling, and myocardial I/R injury animal models (All mice began to undergo experimental surgery when they were 8 weeks old, each experimental group included 8 mice.). Dox-induced DCM group mice were injected with a cumulative dose of 30 mg/kg doxorubicin (Sigma-Aldrich; Merck KGaA) within 30 days after 6 times of intraperitoneal injection (5 mg/kg i.p.). The sham group received the same amount of sterile isotonic saline. Echocardiography was performed 2 weeks after the last injection. To establish Ang II-induced cardiac remodeling model, 5% chloral hydrate (400 mg/kg) was used to anesthetize mice and osmotic pumps (Model 2004, Alzet Scientific Products, USA) supplemented with Ang II (1000 ng/kg/min) or sterile isotonic saline were implanted subcutaneously. Echocardiography was performed 28 days later. To establish myocardial I/R injury model, the mice were anesthetized with the above method, and the left fourth intercostal small incision was made to expose heart. In total, 3 mm from the starting point, the left anterior descending (LAD) coronary artery was ligated and the incision was sutured. The slipknot was released for reperfusion after 45 min. The sham group received the same operation but the LAD were not strangled. Echocardiography was performed 28 days after reperfusion. After echocardiography, the mice were sacrificed immediately after anesthesia, then heart tissues and serum were harvested.

### Cell culture and transfection

H9C2 cardiomyocytes were purchased from the Cell Bank of the Chinese Academy of Sciences (Shanghai, China). H9C2 cells were cultured in DMEM (Gibco, Thermo Fisher Scientific, USA) containing 10% FBS (Gibco, Thermo Fisher Scientific, USA) and 1% penicillin/streptomycin (Gibco, Thermo Fisher Scientific, USA) and placed in incubator at 37 °C with 5% CO_2_.

Small interfering RNA (siRNA) and siRNA negative control (NC) for SLP-2 (siRNA1 SLP-2, sense: 5ʹ-GGUAUGUGCAGAGUCUCAATT-3ʹ, anti-sense: 5ʹ-UUGAGACUCUGCACAUACCTT-3ʹ; siRNA2 SLP-2, sense: 5ʹ-GCAUUAUGGAUCCUUACAATT-3ʹ, anti-sense: 5ʹ-UUGUAAGGAUCCAUAAUGCTT-3ʹ; siRNA3 SLP-2, sense: 5ʹ-CCAGCGAUGUGACAAGUAUTT-3ʹ, anti-sense: 5ʹ-AUACUUGUCACAUCGCUGGTT-3ʹ; siRNA NC, sense: 5ʹ-UUCUCCGAACGUGUCACGUTT-3ʹ, anti-sense: 5ʹ-ACGUGACACGUUCGGAGAATT-3ʹ) were purchased from GenePharma biotech Co., Ltd (Suzhou, China) and transfected into H9C2 cells using Lipofectamine3000 (Invitrogen, Thermo Fisher Scientific, USA). In short, 5 μL siRNA or 5 μL Lipofectamine 3000 were diluted in 62.5 µL Opti-MEM (Gibco, Thermo Fisher Scientific, USA) and incubated at room temperature for 5 min before being mixed and incubated for another 15 min. H9C2 cells were then cultured with the mixture at 37 °C for 4 h and then replaced with fresh preheated DMEM medium for 48 h. The cell proteins were harvested for western blot analysis to select the most effective sequences for subsequent experiments.

To inhibit autophagy, successfully transfected H9C2 cells were pretreated with 10 mM 3-methyladenine (3-MA, MCE, USA) for 4 h, then cultured in DMEM medium containing 1 μM Dox for 24 h, followed by follow-up experiments.

### Echocardiographic evaluation

Mice were anesthetized with 1.5–2.0% isoflurane inhalation, and the cardiac function indexes were measured at the papillary muscle level by using the Vevo2100 instrument (VisualSonics, Canada) with M-mode echocardiography. Finally, left ventricular ejection fraction (EF), left ventricular fractional shortening (FS), left ventricular mass (LV mass), interventricular septum (IVS) thickness, and left ventricular posterior wall (LVPW) thickness were automatically calculated.

### Histological analysis

Mice hearts were immersed in 4% neutral formaldehyde tissue fixative for 24 hours, then dehydrated with ethanole of gradient concentration and xylen, embedded in paraffin, and finally cut into 5μm thick slice. The sections were stained with hematoxylin-eosin (HE), Masson’s trichrome and Picro-Sirius Red (PSR), then imaged under the light microscope to evaluate the myocardial morphology and fibrosis. All histological analysis were performed blind.

### Immunofluorescence (IF) staining

Mice heart tissues were fixed in the frozen section embedding agent (O.C.T., Sakura, USA) and cut into 5μm sections. The sections were dried at room temperature for 30 min and then fixed with 4% neutral formaldehyde tissue fixative for 20 min. After 1×PBS flushing, the tissues were treated with 0.1% TritonX-100 for 5 min and 3% goat serum (ZLI-9022, Beijing Zhongshan Biotechnology) for 1 h, then separately incubated with antibodies against Vimentin (ab8978, abcam, USA), LC3B (ab51520, abcam, USA), PINK1 (23274-1-AP, proteintech, USA) and Parkin (66674-1-Ig, proteintech, USA) at 4 °C overnight, followed by incubation with secondary antibody for 1 h at room temperature. Finally, the nuclei were stained with Hoechst33342. The tissue sections were incubated with wheat germ agglutinin (WGA) working solution (W7024, Invitrogen, USA) at room temperature for 10 min to detect the cardiomyocyte cross-sectional area. The images were observed under a fluorescence microscope.

### Immunohistochemistry (IHC) staining

After dewaxing and rehydration, tissue sections were infiltrated with 3% hydrogen peroxide for 15 min to remove endogenous peroxidase, then the antigen was repaired by using citric acid solution, and incubated with goat serum (ZLI-9022, Beijing Zhongshan Biotechnology) for 1 h to prevent non-specific binding of antibodies. The sections were separately incubated with primary antibodies against Collagen I (14695-1-AP, proteintech, USA), α-SMA (A5228, sigma-aldrich, USA) and Collagen III (22734-1-AP, proteintech, USA) at 4 °C overnight, followed by goat anti-rabbit or anti-mouse IgG (KIT-5004 and KIT-5001, MXB, China) for 1 hour at room temperature. Finally, immunohistochemical reactions were analyzed using a DAB kit. The results of IHC staining were evaluated using ImageJ software. The negative result score of staining intensity was 0, while the low positive, positive or high positive result scores were 1, 2 and 3, respectively. The score of positive staining area <5% was 0, when the area of positive staining area is 5–25%, 26–50%, 51–75% and >75%, scores were 1, 2, 3 and 4, respectively. The final total score is the product of the intensity score and the area score.

### Western blot analysis

The mice left ventricular tissues were lysed with RIPA buffer on ice, and then the protein concentration was measured using a BCA protein assay kit (KGP902, KeyGEN BioTECH, China). Equal amounts of proteins (30 μg) were separated by 8%–12% SDS-PAGE and transferred to PVDF membrane. After sealed with 5% skimmed milk for 1 h at room temperature, membranes were incubated with following primary antibodies overnight at 4 °C: α-SMA (A5228, sigma-aldrich, USA), Collagen I (14695-1-AP, proteintech, USA), Collagen III (22734-1-AP, proteintech, USA), Fibronectin (15613-1-AP, proteintech, USA), GAPDH (HRP-60004, proteintech, USA), Bcl-2 (26593-1-AP, proteintech, USA), Bax (2772 S, cell signaling technology, USA), Cleaved-caspase-3 (9661 S, cell signaling technology, USA), LC3B (ab51520, abcam, USA), Beclin1 (66665-1-Ig, proteintech, USA), ATG5 (12994 S, cell signaling technology, USA), PINK1 (23274-1-AP, proteintech, USA), Parkin (66674-1-Ig, proteintech, USA), Mfn2 (12186-1-AP, proteintech, USA), Drp1 (12957-1-AP, proteintech, USA), p-PI3K (4228 S, cell signaling technology, USA), PI3K (4257 S, cell signaling technology, USA), p-Akt (4060 S, cell signaling technology, USA), Akt (9272 S, cell signaling technology, USA), p-mTOR (2971 S, cell signaling technology, USA) and mTOR (2983 S, cell signaling technology, USA). The membranes were washed 3 times with Tris Buffered Saline with Tween 20 (TBST), and incubated with the secondary antibody at room temperature for 1 h. The protein bands emerged by Immobilon western chemiluminescence HRP substrates (WBKLS0500, Millipore, USA) were photographed using ChemiScope (Clinx Science Instruments, China). The gray value of each band was measured by Chemi analysis software (Clinx Science Instruments, China).

### ROS staining

Levels of intracellular ROS and mitochondrial ROS (mtROS) generation were detected using 2’,7’- dichlorodihydrofluorescein diacetate (DCFH-DA) and MitoSOX (Yeasen, China). The prepared tissue sections were treated with 10 M DCFH-DA for 20 min at 37 °C in the dark, humid chamber. The cell slides were treated with 2 mL MitoSOX red mitochondrial superoxide indicator for 10 min at 37 °C in the dark, humid chamber. The ROS positive counts from four randomly chosen fields observed under a fluorescence microscope were measured.

### TUNEL staining

The tissue sections/cell slides were incubated at room temperature with 0.2% TritonX-100 for 15 minutes, washed with 1×PBS for 3 times and incubated with 1×Equilibration buffer at room temperature for 30 min. Then the sections were incubated with TDT incubation buffer at room temperature for 1 hour. After washing with 1×PBS for 3 times, the nuclei were stained with Hoechst33342. The reagents used are from Vazyme biotech Co., Ltd (Nanjing, China). The images were observed under a fluorescence microscope.

### Determination of ATP content and serum biomarkers

The ATP content and serum biomarkers were measured strictly based on the respective kit instructions (ATP, A095-1-1; AST, C010-2-1; ALT, C009-2-1; TBil, C019-1-1; BUN, C013-2-1; Cre, C011-2-1; MDA, A003-1-1; SOD, A001-3-2, Nanjing Jiancheng Bioengineering Institute, China).

### Evans Blue/TTC Staining

The LAD was re-ligation after I/R surgery, and 2 mL 2% Evans Blue (Sigma-Aldrich, USA) was injected into inferior vena cava. Isolated hearts were frozen at −20 °C for 10 min and cut into five pieces (approximately 1 mm thick) and incubated with 1% 2,3,5-Triphenyltetrazolium chloride (TTC) solution at 37 °C for 20 min. Finally, the heart sections were fixed with 4% neutral formaldehyde for 2 h, and the stained areas were analyzed by ImageJ software.

### Bioinformatics analysis

The proteomic and bioinformatics analyses were performed by Applied Protein Technology Co., Ltd (Shanghai, China).

### Detection of mitochondrial membrane potential

H9C2 cells were incubated with JC-1 working solution (C2006, Beyotime, China) at 37 °C for 20 minutes, then washed with JC-1 buffer for 3 times and observed under fluorescence microscope.

### Statistical analysis

Results are expressed as mean ± standard error of the mean. The Shapiro–Wilk test was used to evaluate the normality of data distribution. Student’s *t* test was used for comparison between the two groups. Multiple group comparisons were performed using 1-way ANOVA, followed by the Tukey test. All statistical analyses were performed with GraphPad Prism 8.0 software (GraphPad Software Inc.). We considered *P* < 0.05 as statistically significant.

## Supplementary information

Supplemental Materials

Original full length western blots

## Data Availability

The datasets used during the study are available from the corresponding author on reasonable request.
