# Supplementary material for: Stomatin-like protein 2 deficiency exacerbates adverse cardiac remodeling
Source: Cell Death Discov. 2023 Feb 14;9:63. doi: 10.1038/s41420-023-01350-z (PMC9929064; doi:10.1038/s41420-023-01350-z)
Supplement: Supplementary file 1 — Supplemental Materials [file 41420_2023_1350_MOESM1_ESM.docx]

**Stomatin-like Protein 2 Deficiency Exacerbates Adverse Cardiac Remodeling**

**Authors**

Yuntao Hu^1^*, Hongwei Jiang^1^*, Yueyue Xu^1^, Ganyi Chen^1^, Rui Fan^2^, Yifei Zhou^1^, Yafeng Liu^1^, Yiwei Yao^1^, Renjie Liu^2^, Wen Chen^1^, Ke Zhang ^3^, Xin Chen^1^, Rui Wang^1#^, Zhibing Qiu^1#^

**Affiliations**

^1^Department of Thoracic and Cardiovascular Surgery, Nanjing First Hospital, Nanjing Medical University, Jiangsu, China

^2^School of Medicine, Southeast University, Jiangsu, China

^3^Department of Thoracic and Cardiovascular Surgery, Changzhou Second People's Hospital, Nanjing Medical University, Jiangsu, China

*These authors contributed equally to this work.

^#^Corresponding author:

Rui Wang

Email: [wr1582@163.com](mailto:wr1582@163.com)

Department of Thoracic and Cardiovascular Surgery, Nanjing First Hospital, Nanjing Medical University, No. 68 Changle Road, Nanjing 210006, China

Zhibing Qiu

Email: [qiuzhibing2009@163.com](mailto:qiuzhibing2009@163.com)

Department of Thoracic and Cardiovascular Surgery, Nanjing First Hospital, Nanjing Medical University, No. 68 Changle Road, Nanjing 210006, China

**Supplemental Figures**


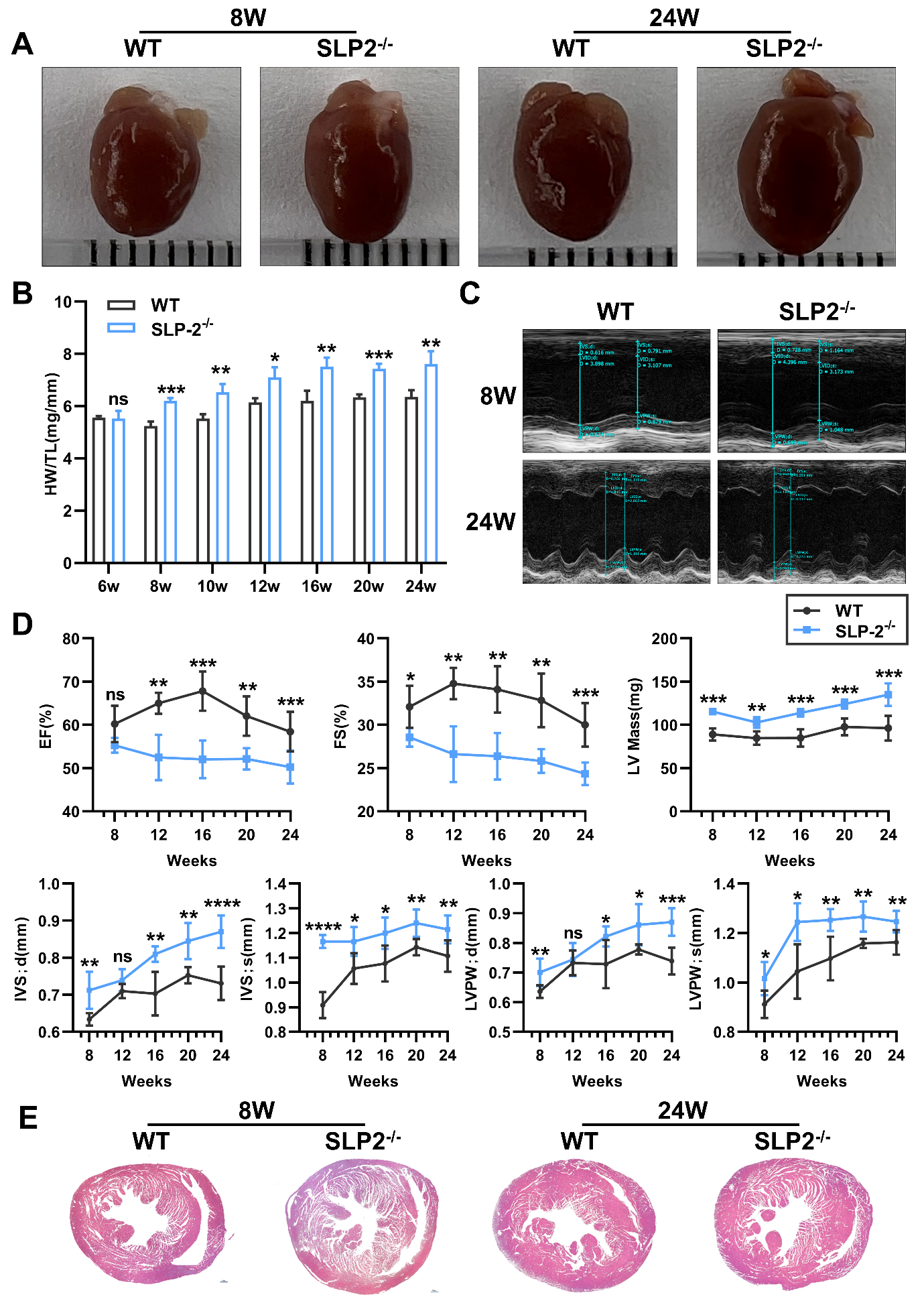


**Figure S1**.

**A**, Representative heart specimens from WT and SLP-2^-/-^ mice at 8w and 24w.

**B**, HW/TL in WT and SLP-2^-/-^ mice at 6w, 8w, 10w, 12w, 16w, 20w and 24w.

**C**, Representative echocardiograms of WT and SLP-2^-/-^ mice at 8w and 24w.

**D**, Echocardiographic parameters (EF, FS, LV mass, IVS and LVPW) of WT and SLP-2^-/-^ mice at 8w, 12w, 16w, 20w and 24w.

**E**, Heart representative images of HE staining in WT and SLP-2^-/-^ mice at 8w and 24w.

(n=3-8 per group, *P<0.05, **P<0.01, ***P<0.001, ****P<0.0001, vs. WT mice, student’s t-test).


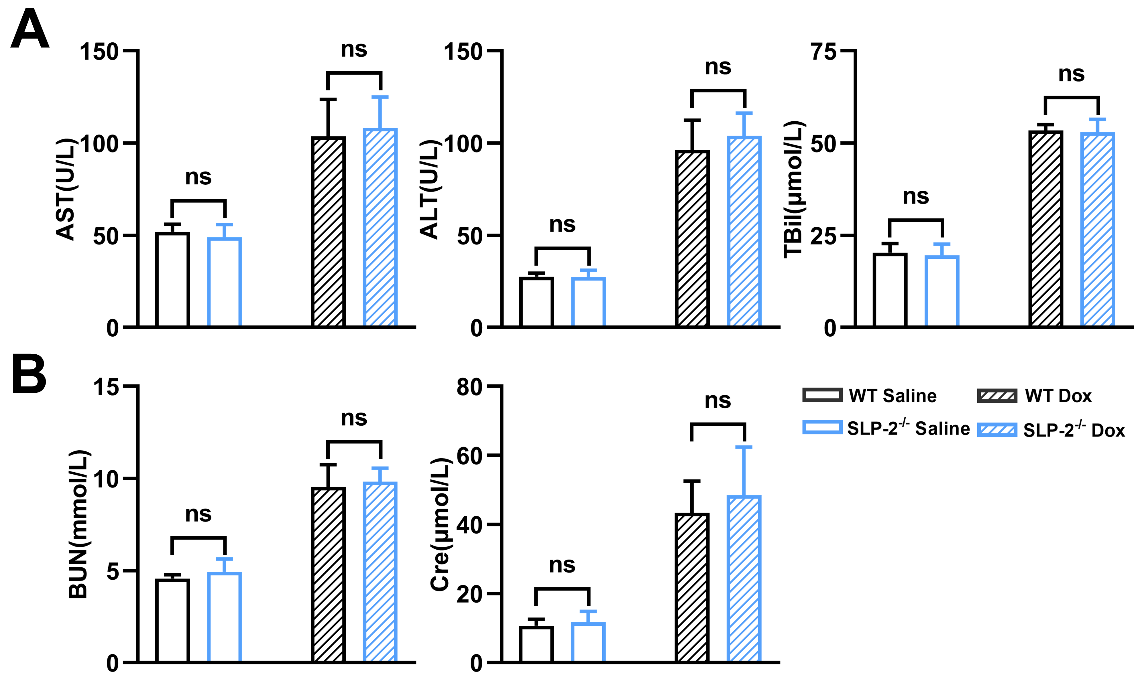


**Figure S2**.

A and B, Serum biochemical indexes of hepatic and renal function in WT and SLP-2^-/-^ mice hearts after saline or Dox treatment.

(n=4 per group, student’s t-test).


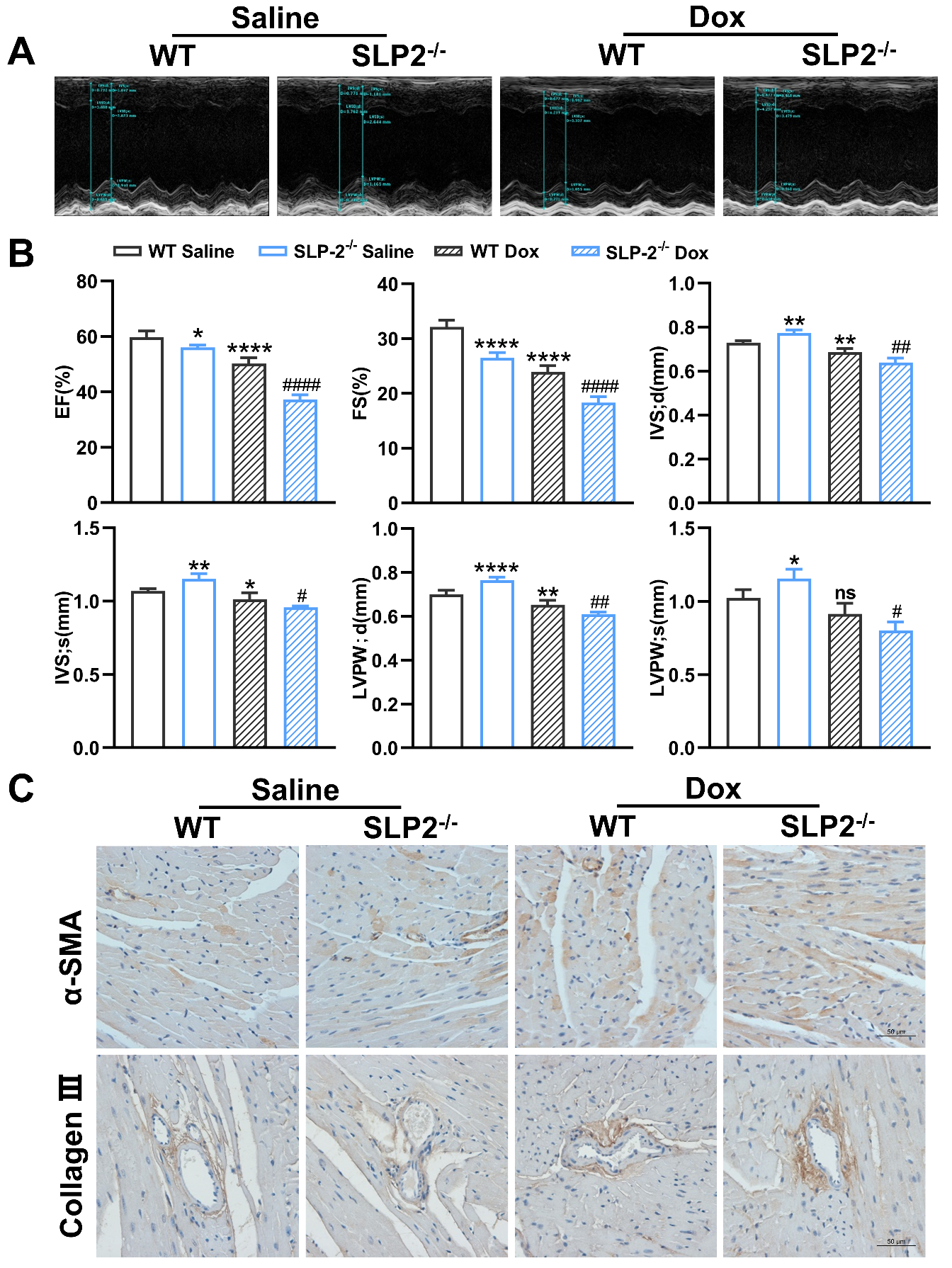


**Figure S3**.

**A** and **B**, Representative echocardiographs and echocardiographic parameters in WT and SLP-2-/- mice after saline or Dox treatment.

**C**, Heart representative images of IHC staining of α-SMA and collagen Ⅲ in WT and SLP-2-/- mice after saline or Dox treatment.

(n=4 per group, *P<0.05, **P<0.01, ***P<0.001, ****P<0.0001 vs. WT Saline group, #P<0.05, ##P<0.01, ###P<0.001, ####P<0.0001 vs. WT Dox group, 1-way ANOVA, Tukey test).


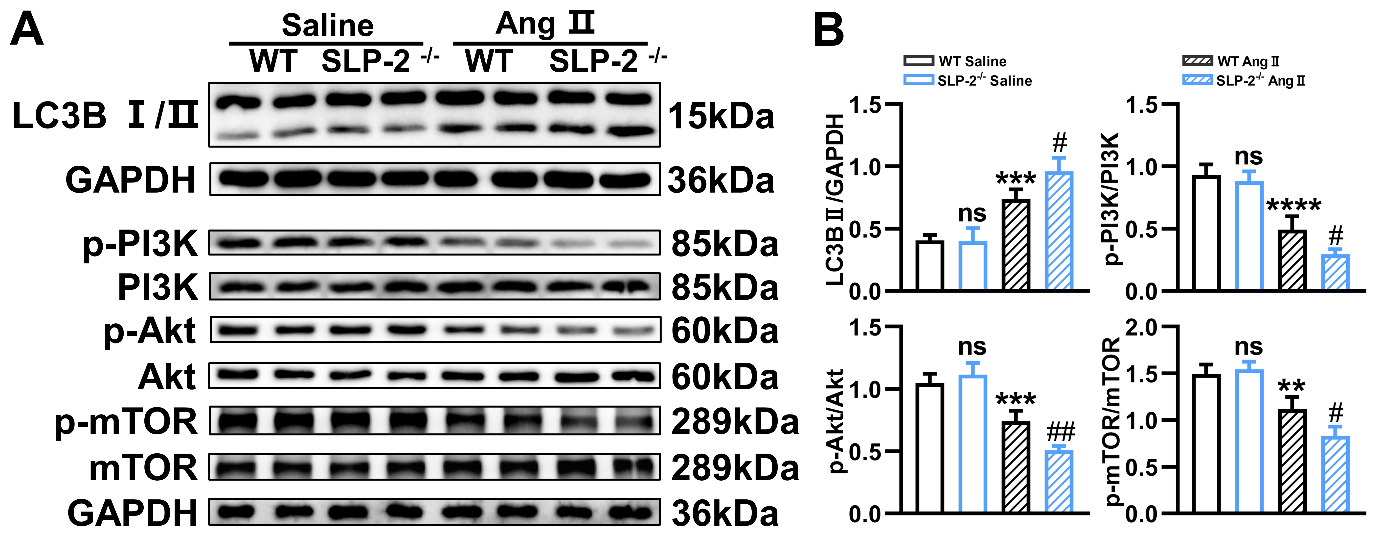


**Figure S4**.

**A** and **B**, Representative images of expression of LC3B, p-PI3K, PI3K, p-Akt, Akt, p-mTOR and mTOR, and quantitative analysis in WT and SLP-2-/- mice hearts after saline or Ang Ⅱ treatment.

(n=4 per group, *P<0.05, **P<0.01, ***P<0.001, ****P<0.0001 vs. WT Saline group, #P<0.05, ##P<0.01 vs. WT Ang Ⅱ group, 1-way ANOVA, Tukey test).


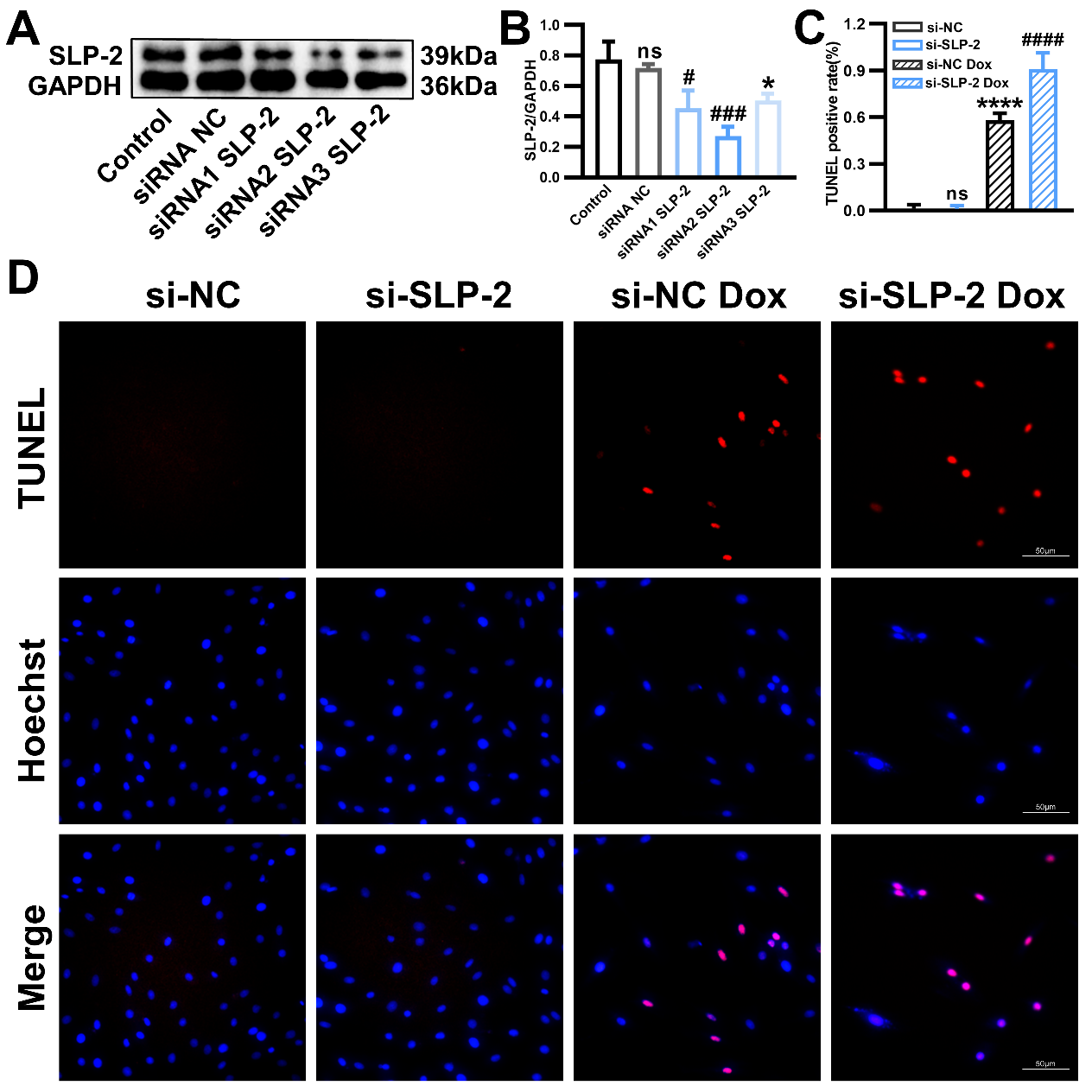


**Figure S5**.

**A** and **B**, Representative images of expression of SLP-2 and quantitative analysis in different groups of H9C2 cells.

**C** and **D**, TUNEL staining and positive rate in different groups of H9C2 cells.

(n=3-4 per group, *P<0.05 vs. Control group, #P<0.05, ##P<0.01, ###P<0.001 vs. siRNA NC group, 1-way ANOVA, Tukey test).


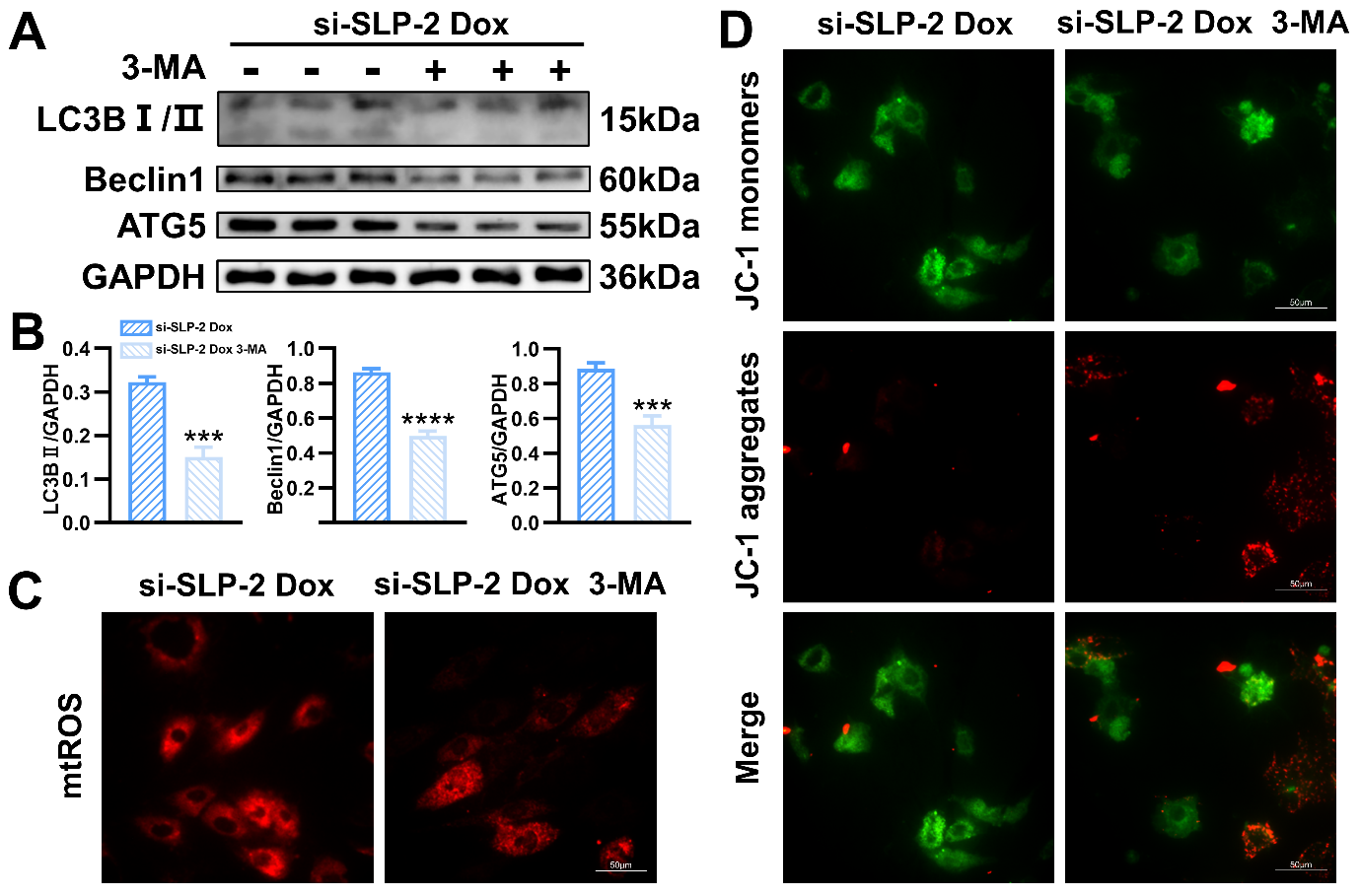


**Figure S6**.

**A** and **B**, Representative images of expression of LC3B, Beclin1 and ATG5, and quantitative analysis in different groups of H9C2 cells.

**C**, Representative images of mitochondrial peroxide staining in different groups of H9C2 cells.

**D**, Representative fluorescence images of JC-1 staining in different groups of H9C2 cells.

(n=3-4 per group, *P<0.05, **P<0.01, ***P<0.001, ****P<0.0001 vs. si-SLP-2 Dox group, student’s t-test).
