# Supplementary figures and images for: Stomatin-like protein 2 deficiency exacerbates adverse cardiac remodeling
Source: Cell Death Discov. 2023 Feb 14;9:63. doi: 10.1038/s41420-023-01350-z (PMC9929064; doi:10.1038/s41420-023-01350-z)

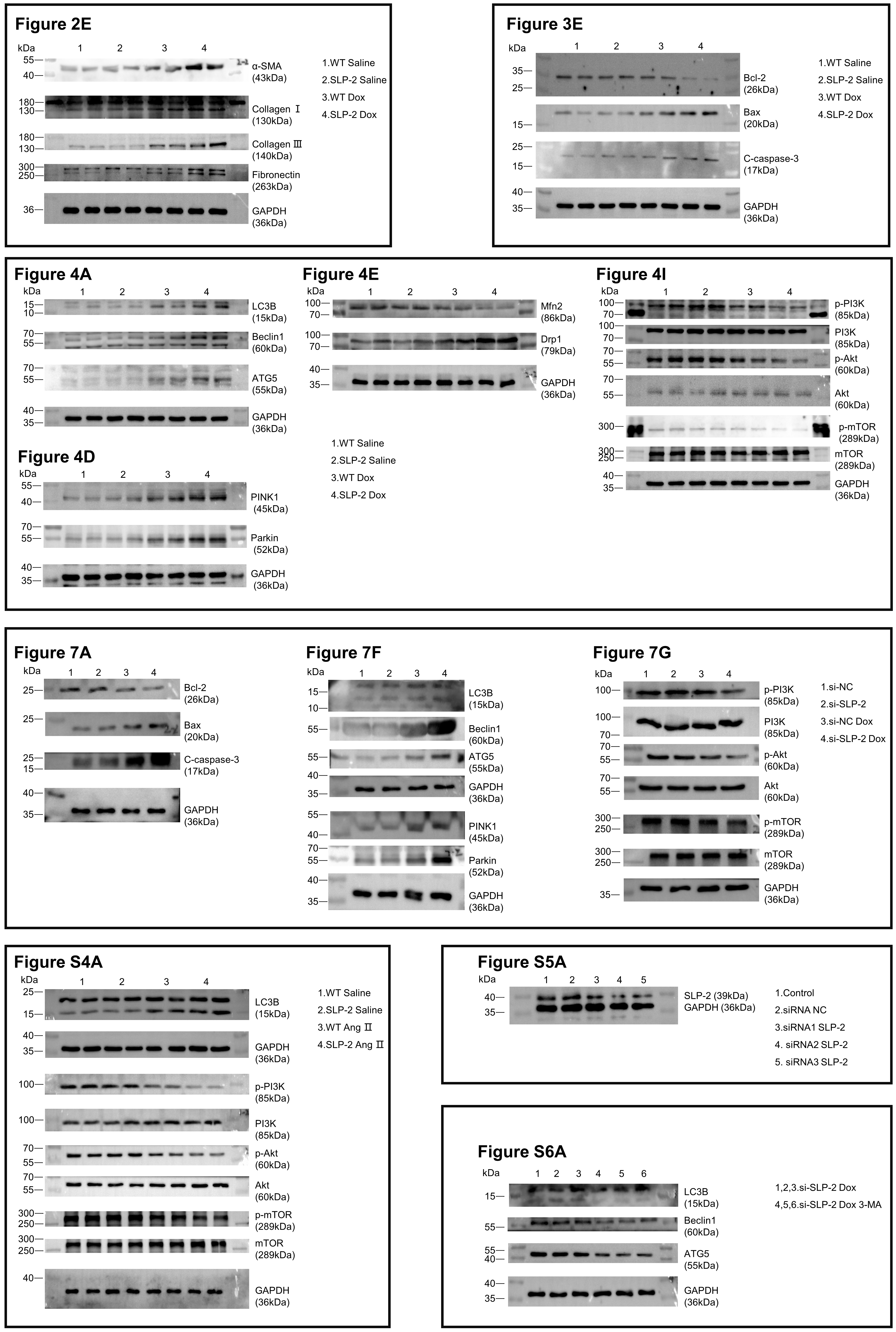

Supplement: Supplementary file 2 — Original full length western blots [file 41420_2023_1350_MOESM2_ESM.tif]
